# Can hearing amplification improve presbyvestibulopathy and/or the risk-to-fall ?

**DOI:** 10.1007/s00405-020-06414-9

**Published:** 2020-10-09

**Authors:** Arne Ernst, Dietmar Basta, Philipp Mittmann, Rainer O. Seidl

**Affiliations:** Department of Otolaryngology at UKB, Charité Med School, Hospital of the Univ of Berlin, Warener Str. 7, 12683 Berlin, Germany

**Keywords:** Presbyvestibulopathy, Hearing aids, Cochlear implants

## Abstract

**Purpose:**

The decline of sensory systems during aging has been widely investigated and several papers have correlated the visual, hearing and vestibular systems and the consequences of their functional degeneration. Hearing loss and presbyvestibulopathy have been found to be positively correlated as is with the risk-to-fall.

**Material and methods:**

The present study was therefore designed as systematic review (due to PRISMA criteria) which should correlate hearing amplification by hearing aids and/or cochlear implants with balance outcome. However, the literature review (Cochrane, PubMed) revealed ten paper (prospective, controlled trials and acute trials) with heterogenous patient popiulations and non-uniform outcome measures (i.e., gait analysis, questionnaires, postural stabilometry) so that no quantitative, statistical analysis could be performed.

**Results:**

The qualitative analysis oft he identified studies showed that hearing amplification in the elderly improves spatio-temporal orientation (particularly with cochlear implants) and that the process of utilizing auditory information for balance control takes some time (i.e., the neuroplasticity-based, learning processes), usually some months in cochlear implantees.

**Discussion:**

Hearing and balance function degenerate independently from each other and large interindividual differences require a separate neurotological examination of each patient. However, hearing amplification is most helpful to improve postural stability, particularly in the elderly. Future research should focus on controlled, prospective clinical trials where a standardized test battery covering the audiological and neurotological profile of each elderly patient pre/post prescription of hearing aids and/or cochlear implantation should be followed up (for at least 1 year) so that also the balance improvements and the risk-to-fall can be reliably assessed (e.g., by mobile posturography and standardized questionnaires, e.g., the DHI).

## Introduction

Presbyvestibulopathy is an age-dependent process of sensory degeneration of the vestibular system which can end up as a clinicially relevant disorder leading to falls. As longitudinal studies of aging have demonstrated, the decline of the sensory systems is inevitable, the most significant correlation can be found (with respect to balance, hearing, vision) with age (BASE) [1]. The Baltimore Study, however, found in a preliminary report (276 participants, mean age 70 yrs) no association between the decline of the sensory systems [2]. A few data only are available on the correlation, prevalence, and time patterns of prebycusis and presbyvestibulopathy development. In a cross-sectional study from the same institution [3], the prevalence for balance dysfunction was given as high as 69% in those over 70 yrs (compared to 63% hearing impairment in this age group, all Caucasians). More specifically, they reported that high-frequency hearing loss (HL) was significantly correlated with saccular, but not utricular or semicircular canal dysfunction (*p* ≤ 0.0001) as evidenced by c/oVEMPs. The authors conclude therefore that screening of high-frequency HL can be utilized as falls screening procedure since saccular dysfunction makes elderly particularly prone to falls [4, 5]. In a systematic review [6], the association between HL, postural control and mobility in older adults was found to be significantly positive between HL and postural control. It confirmed the data of Lin and Ferrucci [7] who reported that people with HL have an increased risk-to-fall in the order of 1.4 × per 10 dB HL (above a 25 dB HL threshold). Older women with poor hearing were as well at higher risks for falls [27]. Another retrospective, propensity matched cohort study (154.414 participants, from 2000–2016, mean age 64 yrs) correlated the HL (defined as first claim for hearing aid dispensing) and accidental falls and found that the 10 yrs-risk attributable to HL was 6.9/100 persons (i.e., a 70 -80% increase compared to normal hearing) [8].

It is therefore the aim of the present review to investigate the impact of diagnosed and treated hearing loss (by hearing aids or hearing implants) on presbyvestibulopathy and, more specifically, the risk-to-fall.

## Material and methods

A systematic literature review was carried out according to the Preferred Reporting Items for systematic Review and Meta-Analyses (PRISMA) [9]. The search should identify studies which describe the incidence, prevalence of auditory and vestibular impairment, the relationship between hearing and balance at different periods of life (adults/elderly), the impact of hearing amplification (hearing aids, cochlear implants) on balance (postural control) and the relationship of the ageing hearing and balance systems (and its consequences).

The search was conducted in PubMed (MEDLINE) and the Cochrane Library on May 2, 2020. Study bibliographies were searched to locate additional material. The keywords used for the search are given in Table 1.

**Table 1:** Search terms used for this systematic review with the obtained search results in PubMed and the Cochrane Library

Search term	Hits/database	
	PubMed	Cochrane Library
Aging and hearing And balance	190	9
Hearing and postural control	707	2
Hearing aids and balance	476	10
Cochlear implants and balance	396	0
Hearing and falls	798	10

### Selection criteria

**Fig. 1:**
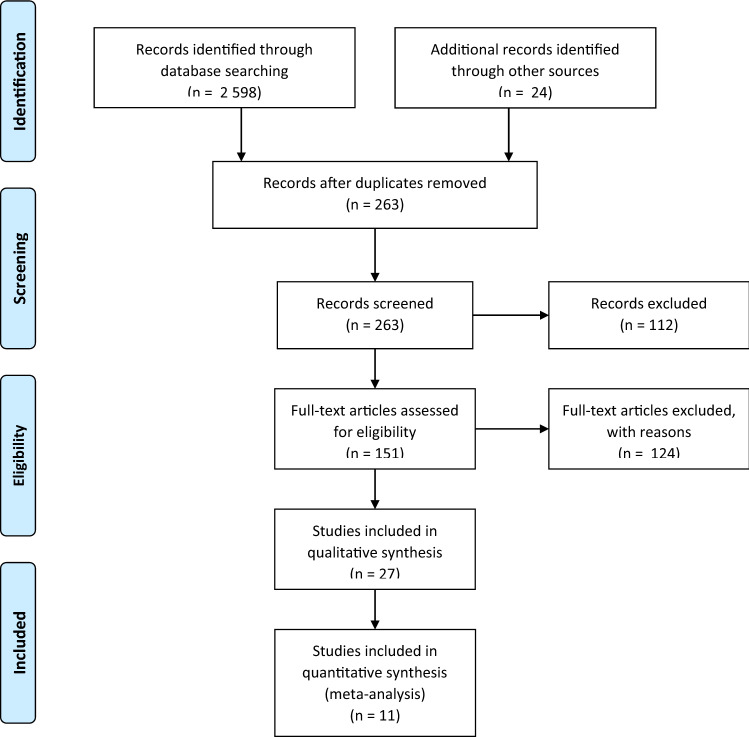
PRISMA flow diagram Moher [9] describing the search strategy and outcome

Prospective, controlled studies (PCS) and acute trials were the only identified study types which were included. No animal studies or reports on hearing and balance disorders in children were considered. After removing duplicated titles, abstracts were screened by two reviewers which then both read the full text if considered eligible for this article (Fig. 1). The PRISMA flowchart was used [9].

### Quality assessment

The methodological quality assessment of appropriate studies was done by two reviewers, independent from one another. The Cochrane Risk-of-Bias tool (RoB) and the Quality Appraisal Checklist for Case Series (Alberta, Canada) were applied and the studies were assessed with respect to patients ‘ selection, type of intervention, outcome measurement and/or incomplete outcome data.

### Data extraction

Study characteristics and outcome data were extracted for each study (Table 2). The following data were sampled: authorship, publication data, type of study, patient characteristics (age, type of disorder, audiological and neurotological diagnostics and/or follow-up), subjective and/or objective outcome measures. Since no uniform outcome measures could be identified (i.e., the studies had variable designs), no data for further statistical meta-analysis could be extracted.

**Table 2:** Synopsis of the selected studies due to PRISMA criteria

Study	Publ year	Study design	Participants	Intervention measurement	Age	Follow-up
Lacerda et al	[10]	PCS, bilateral HA	56	Quest (BBS, SF36, FES-1, BERG	72	Pre fitting/post@4 months
Rumalla et al	[11]	AS, bilateral HA	14	Romberg on foam, quest (BCS), HA on/off with WN	77	n.a
McDaniel et al	[12]	AS, bilateral HA	22	SOT(Equitest) with HA on/off	68	n.a
Negabhan et al	[13]	AS, bilateral HA vs. controls	22 HA/25 con	Group comp, PP (COP), HA on/off	Over 60	n.a
Weaver et al	[14]	AS, HA and bilateral CI	13HA/12CI	Gait analysis (velocity, stride length, swing time)	83HA/55CI	n.a
Parietti-Winkler et al	[15]	PCS, unilateral CI vs. controls	10CI/10cont	SOT (Equitest),pre/post against controls	55/63	1 yr
Louza et al	[16]	PCS, unilateral CI	20	Intraind comparison, SBDT (VertiGuard)	64	Pre/post@3, 5 days
Oikawa et al	[19]	AS, unilateral CI vs. controls	8 CI/8 contr	Group comparison, PP (body sway@COP),on/off	56/24	n.a
Guigou et al	[17]	PCS, uni/bilateral CI vs. control	15uni/7bilCI	Group comparison, dyn post, with/without rot s	53unil/57bil	n.a
Wiszomirska et al	[18]	PCS, unilateral CI	21	Intraind comparison, PP (body sway @ COP)	51 (+ −18)	Pre, post (@3 m)

## Results

The search identified 2 598 results with the strategy outlined in Table 1. After removing duplicates, 263 abstracts were screened and 112 excluded (for several resons, e.g., no interventions, irrelevant populations etc.). 151 full-text articles were assessed and 124 excluded with reasons (no follow-up, incoherent patient population etc.). Finally, out of the 27 studies included in qualitative synthesis, ten were used for the qualitative analysis.

### Hearing aids and balance

Four studies were identified which investigated the influence of hearing aid (HA) use on balance. One study (*n* = 56) was a prospective clinical study (PCS) which used questionnaires to demonstrate the effects of bilateral HA use before and 4 months after the fitting [10]. They showed that the quality-of-life (as measured by the SF-36 questionnaire) was improved and the fear-of-falling reduced, but this population did not have serious balance problems before (average age 77 yrs). The other three studies identified were acute studies which used different outcome measures to report on the effects of HA switched on/off. Rumalla et al. [11] used a Romberg (on foam) and tandem stance test to demonstrate that aided hearing (in the dark) gave better balance results compared to unaided condition (*n* = 14, average age 77 yrs). They did not find any significant relationship between improvement in hearing and balance with their test design.

McDaniel [12] used a dynamic posturography platform (SOT) to compare aided vs. unaided hearing in 22 experienced HA users (a vage 68 yrs) and found no difference in SOT performance. They concluded that randomized, controlled trials (RCT) should be undertaken to get more insights.

Negahban et al. [13] investigated in an acute trial the differences between a group of HA users (*n* = 22) and age-matched controls without HA (*n* = 25) on a static posturography platform with respect to static balance (bilateral sway). They found that HA improve static balance function by reducing the sway velocity.

One other study (acute) used gait analysis (velocity, stride length, swing time etc.) to compare 13 bilateral HA users (mean age 83) with 12 bilateral CI users (aged 55), but found no statistically significant differences which might be due to the large epidemiological and age differences between both groups [14].

### Cochlear implants and balance

The review showed four PCS covering the effects of cochlear implantation on balance.

Parietti-Winkler et al. [15] showed that the balance performance of cochlear implantees (*n* = 10, mean age 55) had come to near-normal values of age-matched healthy controls using a dynamic posturography system. They concluded that the “recovery of auditory information” (by cochlear implantation) could induced the compensatory development of new balance strategies which would explain the improvements.

Louza et al. [16] performed balance assessment before and 3, 5 days after CI, respectively, in 20 implantees (mean age 64 yrs) using a mobile posturography system (VertiGuard) which also calculated the risk-of-fall and found no differences between the pre/post CI performance in this very short follow-up periofd-of-time. This is more than likely since the Parietti-Winkler [23] study demonstrated quite impressively that balance improvement due to neuroplasticity/verstibular compensation/learning mechanisms induced requires at least 6—12 months ‘ follow-up.

Guigou et al. [17] compared uni- (*n* = 15, mean age 53) vs. bilateral (*n* = 7, mean age 57) CI users vs. controls on a dynamic posturography platform using rotating auditory scenes. They found that controls were not affected by those auditory stimuli as well as unilateral CI users (without stereophony), but the rotating sound showed a clear deterioration in the SOT in bilateral CI users. They conclude that the integration of binaural auditory cues in bilateral CI users are important for their balance strategy.

Wiszomirska et al. [18] also investigated body sway on a static posturography platform (intraindividual comparison, *n* = 21, mean age 51) and found at 3 months postop no major differences tot he preop measurements apart from ap sway which was reduced.

The only acute trial in cochlear implantology that we could idenify was a study by Oikawa et al. [19] who investigated postural stability on a static platform (eight experienced unilateral CI users, aged 56) with their systems on/off compared to healthy controls. They found an improvement in all conditions with the CI in operation.

## Discussion

It was the aim to screen all relevant databases (PubMed, Cochrane) for such papers which describe the correlation between hearing amplification (thru HA or CI) and balance outcome. Unfortunately, the few (i.e., 10) papers identified were too heterogenous to be used for a quantitative analysis. The type of study design (PCS/AS, intraindividual comparison, group comparison), patient groups and their characteristics and balance assessment tools (e.g., static/dynamic platforms, gait analysis, questionnaires) were also not enough homogenously to be suited for a meta-analysis. We therefore tried to identify trends and describe mechanisms of interaction between hearing and balance. Two major conclusions can be drawn from most of the publications above: hearing amplification helps to improve spatio-temporal orientation (e.g., 10, 15, 16), particularly with CI and that the process of utilizing auditory information for balance control takes some time (due to the underlying neuroplasticity inducing learning and memory formation) so that the acute studies with the hearing systems on/off did not provide very substantial new information. This is in line with cross-sectional studies [20] which have demonstrated that hearing is an independent predictor of postural balance in the elderly or with the papers from larger longitudinal studies (e.g., Baltimore study, ABC study, US National Health and Nutrition Surveys) [2, 4, 21, 22] which all showed a close correlation between hearing, balance and falls, even if some HL data are based and/or possibly biased by self-reporting only [4]. However, the timing/patterns of this sensory decline is not fully understood in each individual, i.e., there are those elderly with a moderate-to-severe HL, but good balance and vice versa. Gadkaree et al. [2] showed in their sample from the Baltimore study that the sensory functions (balance, vision, hearing) decline independently so that they concluded that other etiological (sensory-specific) features might contribute to this effect which had not yet been shown in the other, previous longitudinal study (BASE) some years ago [1]. The multifactorial input which the vestibular system receives and the dominance of visual cues despite the experimentally proven effects of (good) spatial hearing on maintaining balance are one of those specific interactions between the sensory systems [23, 24]. A similar effect of dissociation between vestibular dysfunction, ageing and presbycusis was described in a cross-sectional study from Singapore (*n* = 216, mean age 60) [25]. This might also be due to ethnic differences between Caucasians, Asians and African Americans since the Jackson Heart Study Cohort reported reduced rates of self-reported HL, dizziness and tinnitus as compared to a Caucasian population [26].

While the phenotype of cochlear hearing loss (i.e., mild, moderate, severe) is neuroanatomically closely correlated to the loss of vestibular and spiral (cochlear) ganglion cells, there is no such correlation to patients with presbycusis [27]. If it comes to the more serious consequences of HL, a meta-analysis revealed that the odds of falling were 2.39 × higher among older adults with HL [28]. Zuniga [29] investigated more specifically the correlation between HL and otolith dysfunction (by c/oVEMP recordings) and found in their cross-sectional study with elderly over 70 a close correlation between high-frequency HL and saccular, but not utricular dyfunction, with noise showing the highest correlation as comorbid factor.

In essence, hearing and balance have proven, close relationship in the degenerative patterns of ageing, but do decline independently as based on interindividual differences. However, hearing amplification is most helpful in elderly to improve postural stability [3]. Future studies should better focus on the medium-term outcome (at least one year) of those audiological/otological interventions (i.e., prescription of hearing aids and/or cochlear implantation) as it is known for the monitoring of cognitive improvements after hearing amplification (e.g., 2, 8). This would require a standardized audiological and neurotological test profile (incl. mobile posturography for daily mobility and questionnaires, e.g., the DHI to cover the subjective self-assessment) done pre and post-intervention (after at least one year). Only controlled, prospective clinical trials (matched with a non-intervention arm) should be able to provide more data on this highly epoidemiologically relevant topic since hearing is the only variable of those ageing sensory systems which can be reliably influenced (i.e., improved) in contrast to vision (macular degernation makes it difficult to fully compensate any deficits) and balance (no vestibular prosthesis in sight replacing the loss of the vestibular receptors). This is one major outcome already of the Baltimore Longitudinal Study and a major NIH research strategy therefore [2].
